# Analysis of uncoated LPGs written in B-Ge doped fiber under proton irradiation for sensing applications at CERN

**DOI:** 10.1038/s41598-020-58049-9

**Published:** 2020-01-28

**Authors:** Gaia Maria Berruti, Patrizio Vaiano, Giuseppe Quero, Tiago Filipe Pimentel Das Neves, Alessandra Boniello, Marco Consales, Paolo Petagna, Andrea Cusano

**Affiliations:** 1 CERN European Organization for Nuclear Research, Experimental Department, Detector Technology Group, CH-1211 Geneva, Switzerland; 2 University of Sannio, Department of Engineering, Optoelectronics Group, I-82100 Benevento, Italy; 3 EPFL École Polytechnique Fédérale de Lausanne, Institute of Electrical Engineering, CH-1015 Lausanne, Switzerland

**Keywords:** Optics and photonics, Optical sensors, Nuclear physics

## Abstract

In this contribution, a complete dissertation concerning the behavior of a Long Period Grating (LPG) inscribed in a B-Ge co-doped optical fiber by means of an excimer laser and exposed to proton irradiation during a recent extensive campaign performed at the European Organization for Nuclear Research (CERN) with a fluence of 4.4·10^15^ p∙cm^−2^ is provided. The experimental results have been thus combined for the first time to the best of our knowledge with numerical simulations in order to estimate the variations of the major parameters affecting the grating response during the ultra-high dose proton exposure. From the correlation between experimental and numerical analysis, the irradiation exposure was found to induce a maximal variation of the core effective refractive index of ~1.61·10^−4^, responsible of a resonance wavelength red shift of ~44 nm in correspondence of the highest absorbed radiation dose of 1.16 MGy. At the same time, a relevant decrease close to ~0.93·10^−4^ in the refractive index modulation pertaining to the grating was estimated, leading to a reduction of the resonant dip visibility of ~12 dB. The effect of the proton beam on the spectral response of the LPG device and on the optical fiber parameters was assessed during the relaxation phases, showing a partial recovery only of the wavelength shift without any relevant change in the dip visibility revealing thus a partial recovery only in the refractive index of the core while the reduction of the refractive index modulation observed during the irradiation remained unchanged.

## Introduction

In the framework of the High Luminosity Large Hadron Collider (HL-LHC) project^1,2^, an upgrade of the most powerful accelerator and particles detectors of the world is foreseen at the European Organization for Nuclear Research (CERN). The novel configuration, planned for 2024, relies on several technological innovations with the aim to further increase the rate and energy of the particle collisions, thus resulting in ten times higher level of radiations, in terms of both ionizing dose and particle fluence. In this context, the development and the integration of new instrumentation for monitoring the ambient parameters, such as temperature, humidity and radiation levels, capable to withstand and operate at radiation doses well exceeding the MGy level and particle fluences above 10^15^ particles/cm^2^, is mandatory for the correct operation of the experiments.

To date, optical fiber-based sensors (OFS) represent an attractive solution to overcome in particular the limitations of the traditional miniaturized capacitive hygrometers^3–5^, due to their radiation resistance^6^, intrinsic electrical insulation, insensitivity to electromagnetic fields and possible application in hostile environments. So far, several studies about the development of humidity sensors based on fiber optic technology have been proposed, recently reviewed in^7^. The use of polyimide coated Fiber Bragg Gratings (FBGs) for relative humidity monitoring in high energy physics experiments at CERN has been successfully demonstrated by our multidisciplinary research group since 2011^8–11^. Afterwards, our investigations moved to the development of a second generation of hygrometers based on metal oxide-coated Long Period Gratings (LPGs)^10,12^. The results collected demonstrated that these innovative devices are characterized by extreme relative humidity sensitivity, particularly below 5 %RH, i.e. where both miniaturized capacitive sensors and polyimide coated FBGs substantially lose accuracy. Nevertheless, to assess their possible application in the new generation of detectors foreseen within the HL-LHC project, a clear understanding of the physical and optical mechanisms involved during the LPGs radiation exposure is mandatory, together with a systematic study of their behavior under realistic radiation levels with respect to those expected at HL-HLC.

Certainly, massive knowledge concerning the effects of irradiation on optical fibers and optical fiber-based devices has been collected over the years, but still not all the factors involved are completely clear and known. In 2018 Girard *et al*. published a very interesting review about the recent advancements on the radiation hardened fiber optic-based systems^13^, highlighting the potential and the future challenges of this innovative technology for the application in harsh environments. In ref. ^13^ the authors also provided an overview of the main radiation-induced effects on several classes of fiber optics and fiber optic-based technology, including both point sensors and distributed sensors. It is well-assessed that radiation alters the fiber properties by creating point defects in silica-based material due to ionization or displacement damage processes leading to structural modifications in the pure or doped amorphous host silica matrix of both fiber core and cladding^14,15^. Therefore, the fiber chemical composition, in terms of dopants and concentrations, plays an important role. Numerous experimental and theoretical studies have been devoted to this topic, most of them are based on the characterization of the structure, optical or electronic properties of the point defects in pure silica^15,16^. On the other side, the number of available studies decreases significantly for Ge-doped silica and furthermore for other dopants relevant for optical fibers. In case of Ge-doped optical fiber, the most important radiation-induce defects, which are responsible for optical absorption bands, are the so-called Ge(1), Ge(2), E’Ge and GeX^17^. The Ge(1) is considered as an unpaired electron trapped on four-fold coordinated Ge atom while the E’Ge defect is formed by an unpaired electron localized on a three-fold coordinated Ge atom. The Ge(2) is considered to be a variant of the Ge(1)^18^. Finally, the structure of the GeX defects is still unknown^15^. As to the radiation-induced effects in B-doped optical fiber, it was found in literature that during γ-irradiation Si-substituted borons trap holes to form boron-oxygen hole centers (B-OHCs). Moreover, trapped electron-type B-E′ centers are created upon irradiation^16^. Point defects are responsible of the two main radiation-induced macroscopic effects such as radiation-induced attenuation and radiation-induced refractive index change which degrade the properties of optical fibers and optical fiber-based sensors when exposed to radiations. The radiation-induced attenuation (RIA), corresponds to an increase of the optical absorption of the fiber^6,13,14,19^. The change of the absorption spectra corresponds to a modification of the refractive index of the fiber, according to the Kramers-Krönig relation^13^, with the magnitude of the attenuation depending on several parameters related to both the irradiation conditions (e.g. dose rate, type of radiation, temperature) and the composition of the fiber under analysis^6,13^. Besides the RIA effect, high energy particles or ionizing radiations were also found to induce an additional refractive index change due to the densification of the glass, via the Lorentz-Lorenz formula^13^.

These effects have been widely investigated in case of FBGs for which it is well-known that the basic characteristics such as peak wavelength, spectral width and amplitude are affected by radiation and that the magnitude of these changes is very dependent on the grating type and fabrication technique^6^. However, in contrast with FBGs, there are only few papers available about LPGs under irradiation, all concerning their response to γ-ionizing particles. An extensive overview of the state-of-the-art can be found in^14,20–22^. The first contribution was provided by Vasiliev *et al*. in 1998 in ref. ^23^, where the response of LPGs written in Ge and N-doped fiber by means of CO_2_ laser was reported, demonstrating that gratings written in Ge doped fibers exhibit high resistance to γ-radiation. In 2013 Kher *et al*. presented the first *in-situ* measurements of the refractive index changes due to a high-level gamma radiation using an LPG inscribed in B-Ge doped fiber through CO_2_ laser^24^. Recently, the effects of mixed neutron and gamma flux on the spectral and sensing response of arc-induced LPGs fabricated in various optical fibers have been presented in^25^, showing that radiations cause only a slight change of the temperature sensitivity of the devices. Combining these experimental results with a numerical model, the same authors provided an estimation of the effect of γ-irradiation in terms of variation of the optical fiber refractive index, clearly depending on the fiber type and composition, for doses up to several hundreds of kGy^25,26^.

The radiation field at HL-LHC will be characterized by the presence of both leptons and hadrons of different kind, masses and energies. Protons seem to be better candidates to simulate the combined effect of Total Ionizing Dose (TID) and Displacement Damage (DD), typical of this field. However, for what concerns the study of the behavior of optical fiber gratings under proton irradiation, only few works about FBGs can be retrieved^27–29^ with a maximum proton radiation dose absorbed by the samples of 100 kGy, while no contribution concerning LPGs has been found in literature. The first experimental results on LPGs subject to proton irradiation have been recently presented by our team in^30^. It should be noted that, in order to decouple the effects of proton irradiation on the LPG from those possibly linked to the presence of the oxide coating required for humidity sensing, therefore establishing a sound reference for future studies, in this initial phase we have decided to focus the attention on uncoated LPGs.

The analysis conducted on the existing literature shows that, even though there is a significant growing interest of the researchers towards the study of these devices applied in radiation environments, the majority of the works published on this topic are limited to the study and comparison of the experimental behavior of fiber optic-based gratings written in several kinds of fibers and with different fabrication techniques in various irradiation conditions. The aim of the present work is to fill up the gap in literature in this sense: to provide a unique and complete dissertation which starts from the analysis of the experimental results and the individuation of the main observables involved in the irradiation process and which allows then to estimate, by a suitable combination of experimental results and numerical simulations, the variations of the major parameters affecting the grating response during the radiations exposure.

The paper was organized accordingly. In the first section, we summarize the main results presented in^30^, collected during the very first proton irradiation campaign of an uncoated LPG inscribed in a single-mode B-Ge co-doped optical fiber, with a broad focus about the LPG resonance wavelength shift and transmitted power variations in both the irradiation exposure and relaxation phases. The next sections are devoted to the explanation of the numerical model applied to correlate the observed spectral changes with the physical and optical parameters related to the LPG such as the refractive index of the optical fiber core in both the regions perturbed and unperturbed during the writing process. Finally, the results of the numerical simulations are discussed.

### On-line monitoring during the irradiation

The irradiation campaign under analysis was performed at the CERN proton irradiation facility, named IRRAD^31^, where a primary beam with a momentum of 24 GeV/c is directly extracted from the Proton Synchrotron accelerator. The details concerning the fabrication of the sample under analysis as well as the description of the experimental irradiation set-up are presented in the section “Methods” at the end of the paper.

**Figure 1:**
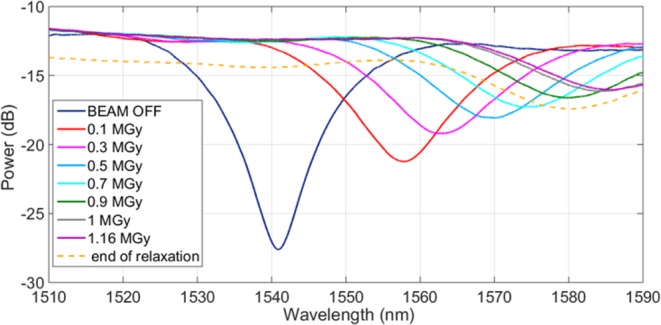
LPG spectral responses during the irradiation experiment.

**Figure 2:**
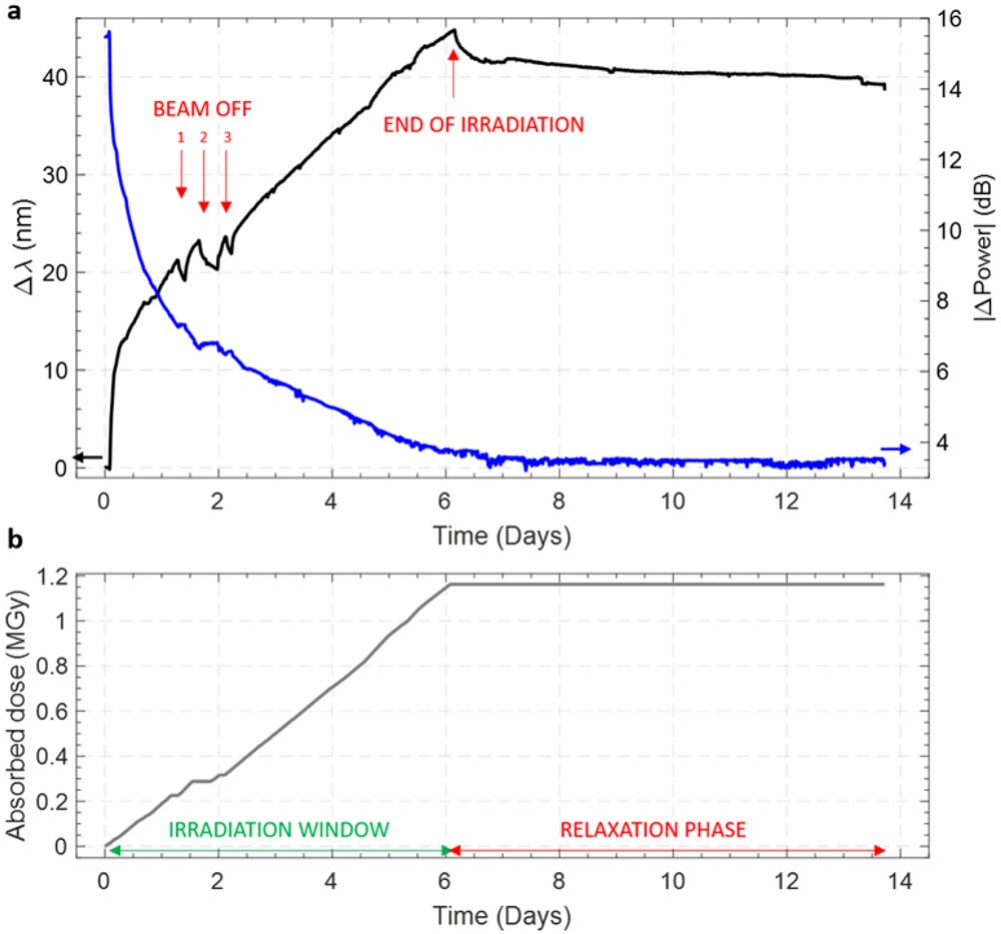
(**a**) LPG resonance wavelength shift (left axis) and visibility changes (right axis) during the full observation period (both irradiation and relaxation phases); **(b)** dose absorbed by the grating during the experiment.

The irradiation experiment consisted in two main stages: in the first 146 hours, the LPG was irradiated up to ~1.16 MGy with an average dose rate of ~2.36 Gy/s. Thereafter, once the proton beam was stopped, the sample was moved out from the beam and the relaxation started. Since we benefited of the exclusive use of the part of the bunker where our sample was installed, we had the opportunity to monitor the response of the sensor in both the over-mentioned phases. Figure 1 provides a synthesis of the spectral evolution of the sample under analysis, starting from its installation in the cavern with the proton beam being off, passing through the beam operation and the exposure to incremental radiation doses, until the end of the relaxation. From the spectral responses of the sensor, acquired with a scanning time of 3 minutes, through a post-processing procedure, we retrieved the variations of the LPG resonant wavelength Δλ and of the spectral dip visibility |ΔPower| = |Power − Power_baseline_|. Results are reported in Fig. 2(a,b), where the two main phases of the data acquisition are well highlighted and related with the dose absorption. As evident, the irradiation was not continuous during our experiment. Indeed, the proton beam was turned off three times for some hours in the first 2 days of acquisition, due to the normal activity of the LHC accelerator. To help in the comprehension of the reported data, we labeled these short breaks as “BEAM OFF” in Fig. 2(a).

Coherently with reported literature for both FBGs^10,14,23,27–29^ and LPGs^14–23^ under irradiation, as soon as the proton beam was started, a shift of the LPG resonance dip towards longer wavelengths was observed, as shown in Fig. 2(a). By the end of the experiment, a final Δλ of ~44 nm was recorded in correspondence of the maximal dose of 1.16 MGy and no saturation phenomenon occurred. This is in contrast with what observed by Taylor *et al*. in^27^ for a lightly Ge-doped silica FBG under protons for which a Bragg wavelength saturation was evaluated at 30 kGy. It should be noted however that in^27^ the irradiation conditions were quite different from our test settings. As a matter of fact, the authors referred to a proton flux four orders of magnitude higher than the one applied during our irradiation, and the maximal dose reached was much lower. It is important to remark that data reported in Fig. 2(a) are not compensated for the effect of the temperature as negligible variations of the order of ±0.2 °C were measured in the IRRAD bunker. Indeed, taking in account the LPG temperature sensitivity of about −0.5 nm/°C, as evaluated during the pre-irradiation characterization of the sample^30^, the above-mentioned temperature variations resulted in a negligible resonance wavelength shift of ~0.1 nm, which is two order of magnitude lower than the total resonance wavelength shift registered at the end of the irradiation.

**Figure 3:**
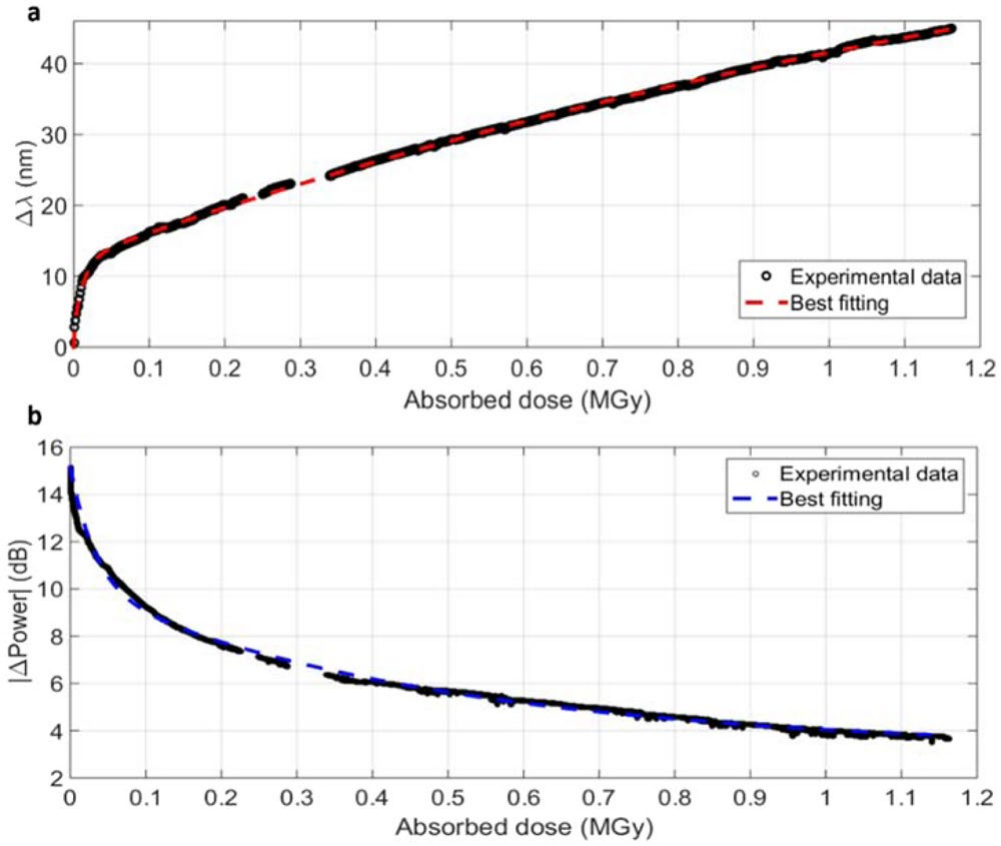
(**a**) LPG resonance wavelength shift and **(b)** visibility changes as a function of the absorbed dose during the irradiation. Data collected during the three short beam breaks were not taken in account in these plots.

In Fig. 3(a) it is shown the irradiation-induced shift as a function of the accumulated dose. An associative exponential fitting defined as the sum of two exponential decays with different time constants, provided the best fit to the experimental data. The parameters of the fitting curve are summarized in Table 1. A two-step behavior of the radiation-induced shift with respect to the dose was observed, with extremely high irradiation sensitivity shown by the sample in the very first hours of the exposure. These results open up solid perspectives for the possible development of reliable ultra-high dose dosimeters based on LPG technology to be applied in the extremely harsh environment of the future accelerators.

**Table 1:** Parameters of the best exponential fitting curves reported in Fig. 3(a,b).

Associative exponential fitting: y = y_0_ + A_1_ * (1 − exp(−x/t_1_)) + A_2_ * (1−exp(−x/t_2_))						
	y_0_	A_1_	t_1_	A_2_	t_2_	R^2^
*Δλ vs*. *Absorbed dose*	0.65899	11.55319	0.01059	63.76648	1.621716	0.99
*|ΔPower| vs*. *Absorbed dose*	15.1544	−5.06094	0.033165	−6.915	0.481976	0.99

As to the effect of the radiations on the LPG dip visibility, we observed a gradual and significant decrease of the power, for a total reduction of 12 dB in correspondence of the highest absorbed dose, as results from Fig. 2(a). Figure 3(b) shows the attenuation of the optical transmitted power with respect to the gradual absorption of dose by the sample. Similarly to the *Δλ vs*. *Absorbed dose* curve, the same fitting function was successfully applied to the |ΔPower|, as shown in Table 1.

Once the proton beam was turned off, we observed a reversed direction of the Δλ (e.g shift towards lower wavelengths), as evident from Fig. 2(a). A post-irradiation recovery shift of ~6 nm was measured after 7.5 days from the interruption of the beam. This corresponds to a recovery of the 14% of the total wavelength shift experienced during the full irradiation. On the other hand, no noticeable recovery in the LPG transmitted power occurred.

**Figure 4:**
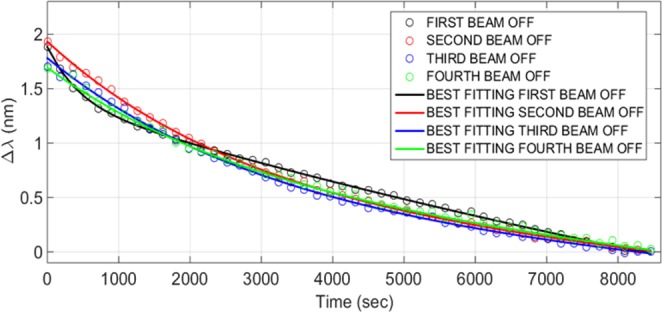
LPG relaxation phase during the very first 2.5 hours of the 4 stops of the proton beam in comparison. The choice of this observation time window corresponds to the duration of the shortest beam stop (the third OFF reported in Fig. 2(a)).

In Fig. 4 the LPG relaxation data collected during the very first 2.5 hours of every proton beam stop are shown for the sake of comparison. The first three data sets refer to the recovery of the sensor during the three short beam breaks registered in the first two days of the data acquisition, due to the LHC normal operation. The fourth data set corresponds to the LPG recovery at the very end of our irradiation experiment. As evident, in the abovementioned four beam interruptions, the sample LPG did not show the same dynamics. As a matter of fact, during the first beam stop, a fast Δλ decrease in the first minutes of the relaxation was followed by a slower dynamic towards the end of the observation period. An associative exponential fitting was applied to match the experimental results collected during the first beam interruption while in case of the last three interruptions, a single constant time exponential model with constant times quite similar to each other was sufficient to correctly fit the experimental data. The parameters of the exponential fittings applied to the abovementioned data sets of Fig. 4 are reported in Table 2.

**Table 2:** Parameters of the best fitting curves reported in Fig. 4.

Associative exponential fitting: y = y_0_ + A_1_ * (1 − exp(−x/t_1_)) + A_2_ * (1 − exp(−x/t_2_))						
	y_0_	A_1_	t_1_	A_2_	t_2_	R^2^
First beam OFF	1.8818	−4.0420	19993.3852	−0.5029	430.7403	0.99
Second beam OFF	−0.2022	2.1064	0.382423	0	—	0.99
Third beam OFF	−0.2628	2.0048	0.475601	0	—	0.99
Fourth beam OFF	−0.2243	1.9177	0.441618	0	—	0.99

### Numerical simulations and discussion

In order to correlate the experimental results shown in the previous section with the dose effects on the main physical parameters pertaining to the LPG device, an extensive numerical analysis has been carried out.

This section is divided in three main parts: the first one relies on the description of the theoretical and numerical model used for the simulation of LPGs; the second one pertains to the analysis of the dependence of the LPG spectrum on the physical parameters of the fiber, which are expected to change with the dose; finally, the third part is devoted to the correlation between the experimental changes in the spectrum observed during and after the protons exposure and the modifications in the physical parameters of the fiber expected from the numerical model.

### Theoretical model

Over the years, our research group has developed a powerful, highly versatile environment for the design and simulation of LPG devices that can provide excellent matching between numerical and experimental results. Our simulation tool, implemented in MATLAB® programming language, is based on the coupled-mode theory^32,33^ and the LP approximation is used to solve the modes within a cylindrical dielectric waveguide^34^. The algorithm proceeds through successive steps, after which the solution becomes progressively more precise.

The first step is the calculation of the propagation constants of the fundamental core mode and cladding modes. These provide a first estimate of the spectral location of the excited resonances, through the well-known phase-matching relation^32^

1 $${{\rm{\lambda }}}_{{\rm{res}},{\rm{j}}}=({{\rm{n}}}_{{\rm{eff}},01}-{{\rm{n}}}_{{\rm{eff}},0{\rm{j}}})\cdot \Lambda $$

where λ_res,j_ is the resonant wavelength corresponding to the excitation of the *j*th order cladding mode, n_eff,01_ and n_eff,0j_ are the effective refractive indexes of the fundamental core and the excited cladding modes, respectively, while Λ is the grating period.

This is followed by the calculation of the coupling coefficients, which allows improving the estimation of the resonance wavelength according to the modified Bragg condition^33^:

2 $$\frac{2{\rm{\pi }}}{{\rm{\lambda }}}\cdot ({{\rm{n}}}_{{\rm{eff}},01}-{{\rm{n}}}_{{\rm{eff}},0{\rm{j}}})+{{\rm{s}}}_{0}\cdot ({{\rm{\zeta }}}_{01,01}({\rm{\lambda }})-{{\rm{\zeta }}}_{0{\rm{j}},0{\rm{j}}}({\rm{\lambda }}))=\frac{2{\rm{\pi }}}{\Lambda }$$

Here, s_0_ is the coefficient of the first Fourier component of the sinusoidal grating, while ζ_01,01_ and ζ_0j,0j_ are the self-coupling coefficients of the core and the *j*th cladding modes, respectively.

The coupling coefficients also help to determine the value of the transmittance i.e. the power transferred from the fundamental core mode to that of the cladding, at the resonant wavelength^35^:

3 $${{\rm{T}}}_{0,{\rm{j}}}={\cos }^{2}({{\rm{\kappa }}}_{01,0{\rm{j}}}\cdot {\rm{L}})$$

In this relation, L is the grating length, and k_01,0j_ is the coupling coefficient for the *j*th cladding mode, which is a function of the overlap integral of the core and cladding modes and of the refractive index modulation induced by the writing process.

Finally, the coupled modes differential equations are solved for the calculation of the full transmission spectrum.

By adopting this numerical model, various LPG configurations have been demonstrated by our group, including single^36^ or double^37^ coated LPGs operating in modal transition and the combination of the dispersion turning point and the modal transition^38^.

### Analysis of the dependence of the spectrum on the physical parameters of the fiber

The LPG under investigation was written in a photosensitive single-mode B-Ge co-doped optical fiber PS1250/1500 provided by FiberCore, featuring a cladding diameter of 125 µm, mode field diameter between 8.8 µm and 10.6 µm at 1550 nm and numerical aperture of 0.12–0.14. The refractive indices of the core and the cladding are 1.4498 and 1.4440, respectively. These values are referred to a wavelength of 1550 nm and dispersion is not taken into account. A grating period of 308 µm was used in order to excite the cladding mode LP_0,8_ with a resonance at around 1540 nm. The grating length was set to 2.36 cm and the spectrum was evaluated in air. In order to obtain a perfect match between experimental spectrum and numerical simulation, a core refractive index modulation depth (Δn_co_), defined as the difference between the refractive index of the core in the regions written by UV (n_co_,_high_) and the one related to the non-perturbed regions of the core (n_co_,_low_), of 2.55·10^–4^ was found.

**Figure 5:**
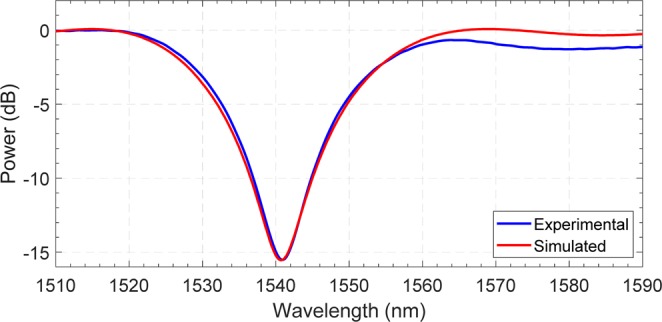
Comparison between the experimental pre-irradiation LPG spectrum at room temperature and that obtained from numerical simulation.

The comparison between the measured pre-irradiation spectral response of the LPG sample at room temperature and the spectrum obtained from simulation is shown in Fig. 5.

From previous studies reported in literature, the exposure to radiation of an LPG device is expected to induce two main effects, namely the RIA and radiation-induced silica densification^14,24^. Therefore, two main parameters pertaining to optical fibers are affected by ionizing radiation: the optical fiber losses and core refractive index^14,24^. Nevertheless, the limited length of the grating-based devices (up to few centimeters) allows us to neglect the optical losses effects and to focus the attention exclusively on the changes induced on both n_co,low_ and n_co,high_. The variation of other parameters, including the grating period and the refractive index of the cladding, is neglected according to previous works^24,26^.

**Figure 6:**
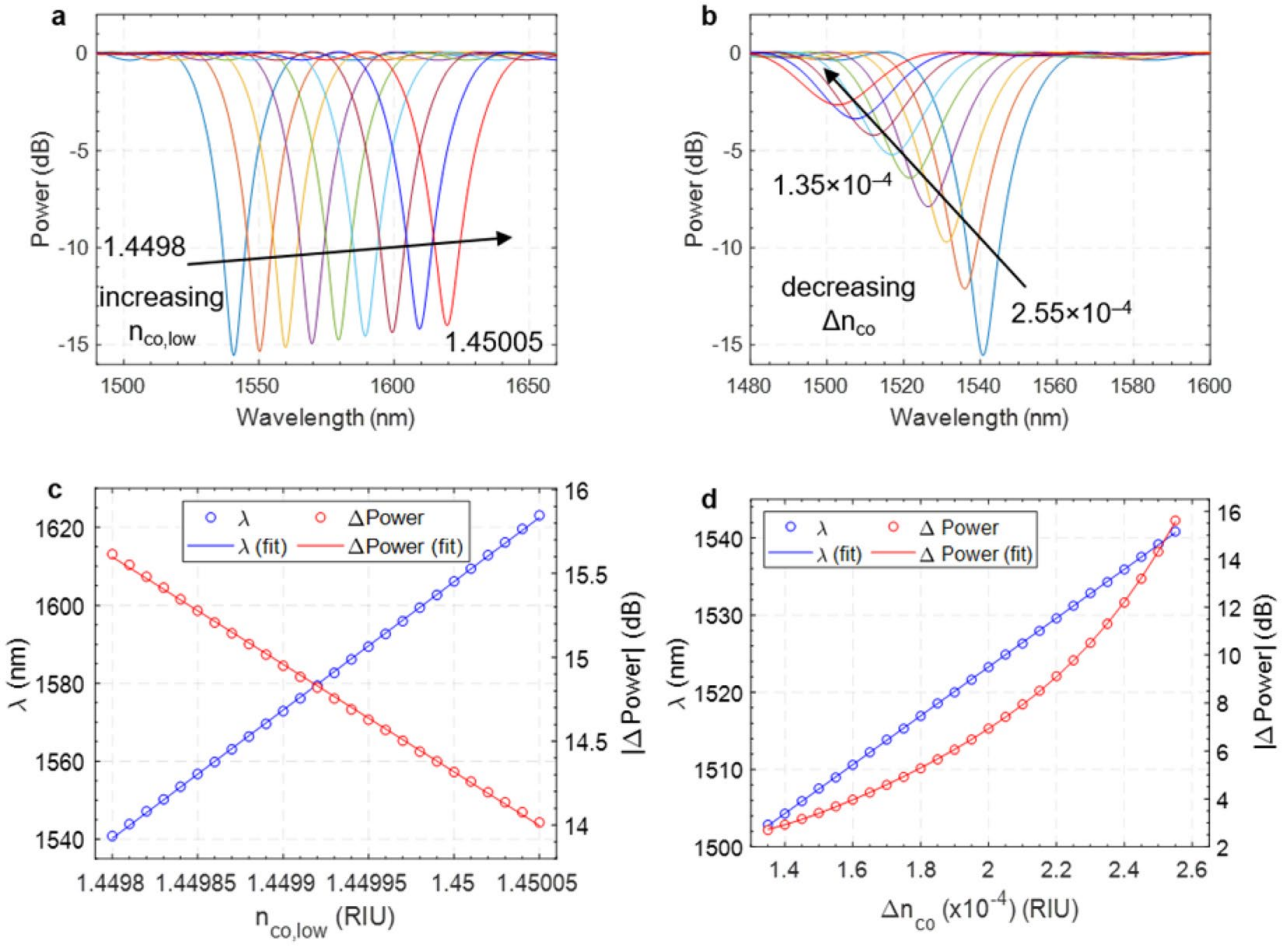
Results from numerical simulations: LPG spectra evaluated after **(a)** a positive variation of n_co,low_ in the range 1.44980 ÷ 1.45005 with a step of 1·10^–5^ and **(b)** a negative variation of Δn_co_ in the range 1.35·10^−4^ ÷ 2.55·10^−4^ with a step of 0.5·10^–5^. Resonance wavelength and visibility and their corresponding fitting functions due to changes in **(c)** n_co,low_ and **(d)** Δn_co_.

Specifically, the effect of a numerically simulated perturbation of n_co,low_ on the LPG spectrum, with Δn_co_ fixed to the pre-irradiation value of 2.55·10^–4^, is reported in Fig. 6(a). An increase of n_co,low_ leads to a red shift of λ_res_ with a linear behavior and a sensitivity of 3.292·10^5^ nm/RIU, coupled to a small linear decrease of |ΔPower| with a sensitivity of -6405 dB/RIU, as shown in Fig. 6(c). In particular, the trend obtained for λ_res_ is clearly consistent with the one described by Eqs. (1) and (2). On the contrary, a decrease of Δn_co_, with n_co,low_ fixed to its nominal value of 1.4498, leads in the simulations to a blue shift of λ_res_ and a significant decrease of |ΔPower|, as evidenced in Fig. 6(b). Specifically, a sensitivity of 3.164·10^5^ nm/RIU was found for λ_res_, while a polynomial dependence (of the fourth degree) is found for |ΔPower|, as reported in Fig. 6(d). These trends find their confirmation in Eqs. (1) and (3), respectively.

### Numerical-experimental correlation to derive the physical variations induced in the fiber by the dose

**Figure 7:**
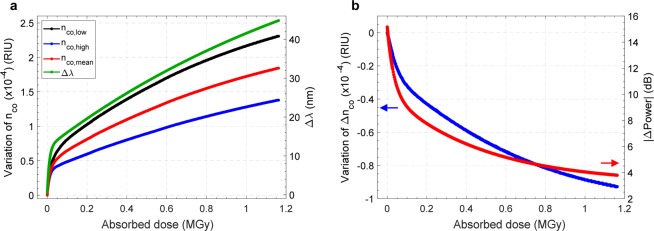
Variations of **(a)** n_co_ (on the left axis) and of Δλ (on the right axis), **(b)** Δn_co_ (on the left axis) and |ΔPower| (on the left axis) as a function of the absorbed dose. In both the plots, for each data set, the corresponding fitting curves are also reported.

After this first step, focusing on a separated evaluation of the relationships between the transmission spectrum and the physical parameters of the device susceptible to changes induced by the proton beam exposure, a further numerical analysis has been devoted to assess the LPG spectral behavior (i.e. λ_res_ and |ΔPower|) when both n_co,low_ and Δn_co_ are considered as simultaneously changing variables. The results obtained from this multi-parametric simulations were then compared with the experimental trends of λ_res_ and |ΔPower| shown in Fig. 3(a,b) with the aim of retrieve the evolution of the core refractive indexes (n_co,low_, n_co,high_, n_co,mean_ = (n_co,low_ + n_co,high_)/2) and Δn_co_ as a function of the dose, as reported in Fig. 7. For this class of parameters as well, the function that best describes the results reported in Fig. 7 is an associative exponential fitting, whose coefficients are shown in Table 3. As expected and in agreement with the literature^14,24^, the absorbed dose leads to an increase of the core refractive index, affecting both n_co,low_ and n_co,high_. Focusing on n_co,mean_, a maximum variation of ~1.88·10^−4^ was estimated in correspondence of the highest absorbed radiation dose. This effect has a major role in determining the dose-induced wavelength shift observed during the irradiation tests, as also confirmed by the remarkable similarity between the *Δλ vs*. *Absorbed dose* and *n*_*co*,*mean*_ *vs*. *Absorbed dose* curves, as a clear consequence of Eq. (1).

**Table 3:** Parameters of the best exponential fitting curves reported in Figs. 7 and 8.

Associative exponential fitting: y = y_0_ + A_1_ * (1 − exp(−x/t_1_)) + A_2_ * (1 − exp(−x/t_2_))						
	y_0_	A_1_	t_1_	A_2_	t_2_	R^2^
(Δn_co,low_, Absorbed dose)	0.02606	0.5435	0.01857	2.587	1.041	0.99
(Δn_co,high_, Absorbed dose)	0.001625	1.915	1.534	0.3622	0.01057	1
(Δn_co,mean_, Absorbed dose)	0.01703	2.19	1.168	0.4468	0.01485	0.99
(Δ(Δn_co_), Absorbed dose)	0.009602	−0.91	0.7678	−0.2324	0.04016	0.99
(Δn_co,eff_, Absorbed dose)	0.01797	0.377	0.01858	1.823	1.054	0.99
(Δn_cl,eff_, Absorbed dose)	0.0005503	0.01132	0.01853	0.05172	1.01	0.99

As a matter of fact, the mean core refractive index depends on the increase of the refractive index in both the unperturbed and perturbed areas. Moreover, during the irradiation exposure, we have observed a reduction in |ΔPower|, which can be correlated to a decrease of the modulation strength of the grating (Δn_co_). Specifically, the perturbed regions (n_co,high_) have a lower sensitivity to radiation due to the previous exposure to UV during the fabrication process, while the unperturbed regions (n_co_low_) show a higher sensitivity. A reduction in the grating modulation depth Δn_co_ of ~0.93·10^−4^ was estimated in correspondence of the highest absorbed radiation dose, as results from Fig. 7(b). A similar behaviour was observed in previous works^39,40^ where, in addition to the radiation-induced Bragg wavelength shift, a reduction in the reflectivity was also reported. Here, according to the authors, the sections of the fiber core that received different UV-fluences during grating inscription possess different sensitivities to radiation^39^.

Since the position of the resonance of an LPG is governed by the phase-matching condition (Eq. (1)), which depends directly on the effective refractive indices of the core and the excited cladding mode, the variation of these parameters as a function of the absorbed dose was evaluated according to the expressions reported below^30^:

4 $${{\rm{n}}}_{{\rm{co}},{\rm{eff}}}({\rm{\lambda }})=\frac{{{\rm{\beta }}}_{{\rm{co}}}({\rm{\lambda }})\cdot {\rm{\lambda }}}{2{\rm{\pi }}}$$

5 $${{\rm{n}}}_{{\rm{cl}},{\rm{eff}}}({\rm{\lambda }})=\frac{{{\rm{\beta }}}_{{\rm{cl}}}({\rm{\lambda }})\cdot {\rm{\lambda }}}{2{\rm{\pi }}}$$

where β_co_(λ) and β_cl_(λ) are the propagation constants of the core and the LP_0,8_ cladding mode, respectively.

**Figure 8:**
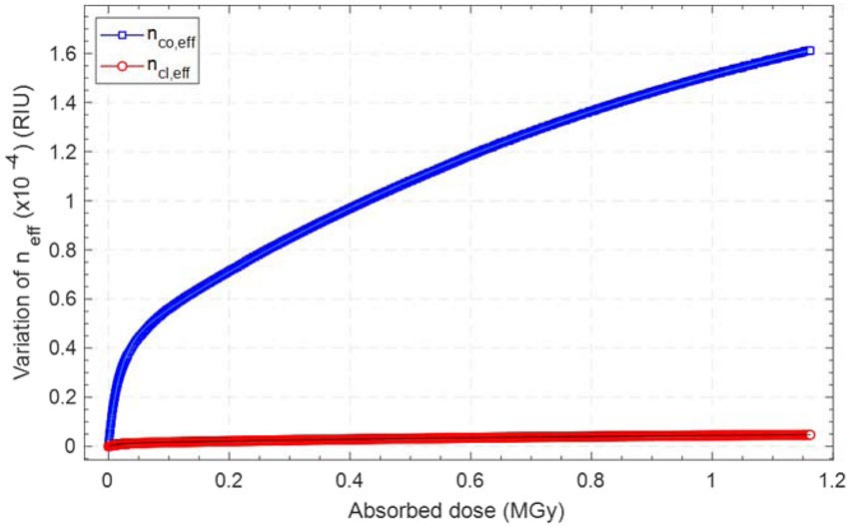
Dose calibration curve related to the core and cladding effective refractive indices evaluated by merging numerical simulations and experimental data.

As shown in Fig. 8, a maximum variation of 1.61·10^−4^ RIU was evaluated for n_co,eff_, while the n_cl,eff_ referred to the LP_0,8_ cladding mode was found to increase only of 4.72·10^−6^ RIU, in agreement with the results reported by Kher *et al*. in^24^.

**Figure 9:**
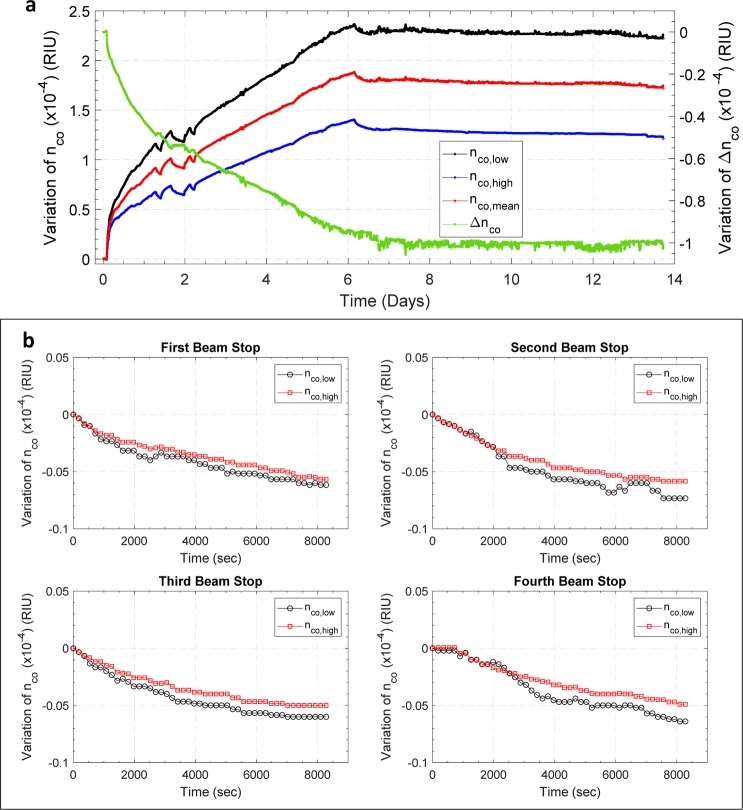
(**a**) Time variations of n_co,low_, n_co,high_ and n_co,mean_ (left axis) and of Δn_co_ (right axis) during the whole LPG spectrum acquisition campaign. These trends were evaluated by merging multi-parametric simulations and experimental data. **(b)** Extended view of the time variation of n_co,low_ and n_co,high_ occurred during the very first 2.5 hours of the 4 stops of the proton beam.

The numerical analysis has been also extended to the evolution of the LPG spectrum due to beam-stops experienced during the irradiation tests and to the relaxation undergone after the end of the irradiation, reported in Fig. 2(a). In this case, the aim of the analysis was to understand how the physical parameters of the LPG device changed as the proton beam was turned off. In particular, we compared the time variations of λ_res_ and |ΔPower| with those obtained by a multi-parametric simulation involving a simultaneous modification of both n_co,low_ and Δn_co_. As a result, the calculated variation of n_co,low_, n_co,high_, n_co,mean_ and Δn_co_ as a function of time is reported in Fig. 9(a). In addition, Fig. 9(b) shows a zoomed view of the time variation of n_co,low_ and n_co,high_ occurred during the very first 2.5 hours of the 4 stops of the proton beam, similarly to the measured values of Δλ reported in Fig. 4. The main evidence is the partial recovery pertaining to n_co,mean_ of 0.05 ÷ 0.07 ·10^−4^ after each beam stop, including the end of the irradiation. However, differently from the beam-on phases simulation shown in Fig. 9, here the recovery featured the same variation of both n_co,low_ and n_co,high_ revealing a relaxation phenomenon occurring at constant modulation depth. This behavior is consistent with the blue wavelength shift and the negligible visibility changes observed in the beam-off phases and in the final post-exposure relaxation.

### Conclusions and outlook

In this work we provide a follow-up analysis of our recent investigations conducted for the first time to analyze the spectral behavior of uncoated LPGs inscribed in a commercial photosensitive single-mode B-Ge co-doped optical fiber exposed to a proton fluence of 4.4·10^15^ p∙cm^−2^ for a total absorbed dose of 1.16 MGy.

The aim of the present work was to correlate the experimental results, including wavelength shift and dip visibility changes, with the effects of proton irradiation on the main physical parameters pertaining to the LPG device, such as the optical fiber core refractive index and the core refractive index modulation.

In particular, we found that the radiation-induced increase of the core refractive index in the unperturbed regions of the fiber is the main responsible of the wavelength red shift observed during the experiment, while the decrease of the LPG resonant depth is correlated to a reduction of the core refractive index modulation in the fiber portions perturbed during the fabrication of the grating. More specifically, the irradiation exposure induced a maximal variation of the core effective refractive index of the LPG device of ~1.61·10^−4^ and a decrease of the modulation of the core refractive index of ~0.93·10^−4^ in correspondence of the highest absorbed radiation dose of 1.16 MGy, in agreement with previous studies reported in literature, limited to the effects of radiations on LPGs.

Moreover, the time variations of the optical parameters of the LPG device showed that during the beam off phases and in the final relaxation at the end of the irradiation campaign, a partial recovery of n_co,mean_ was recorded in correspondence of a blue shift of the resonant wavelength, while no variations in the core refractive index modulation were observed, coherently with the invariance of |ΔPower|.

In particular, after 7.5 days from the end of the exposure, a recovery of 0.15·10^−4^ was recorded for n_co,mean_ in correspondence of a blue shift of the resonant wavelength of about 6 nm with negligible changes in core refractive index modulation as a consequence of the minimal variations in the transmitted power.

The results presented in this paper pave the way for the development of a novel class of miniaturized optical dosimeters potentially providing a single device with high sensitivity from very low to ultra-high ionizing doses. Suitable compensation schemes have to be be judiciously designed in order to decouple the radiation-induced effects from other environmental factors (temperature, humidity in case of coated devices, just to name a few examples). Accurate investigations are currently running to verify if wavelength shift and visibility changes may be opportunely combined to provide a temperature self-referenced dosimeter platform without requiring any external thermal compensation. This might have a very positive impact on the complex task of radiation monitoring in future high energy physics detectors, where the state-of-the art approach for on-line radiation monitoring is represented today by the CERN RadMon^41^ system. As a comparison, this system, developed for the challenges at LHC, is a multi-sensor system gathering in a volume of the order of 20 × 20 × 10 cm^3^ a set of three RadFET dosimeters for TID measurement, three silicon diodes for DD monitoring, and SRAM memories to account for High Energy Hadrons and thermal neutrons.

In view of the on-going development of coated LPG-based hygrometers for CERN experiments, this study will be complemented through a detailed experimental campaign devoted to assessing the effects of the humidity-sensitive coating on the spectral changes observed during the absorption of strong doses of radiation. Moreover, it will be interesting to investigate the radiation-induced response of several types of LPGs, written using different kinds of fabrication techniques. In this regard a series of dedicated irradiation campaigns at high irradiation doses will be launched in order to estimate the variations of the major parameters affecting the response of different types of gratings (excimer, arc-induced, femtosecond laser LPGs) during the radiations exposure.

Finally, it is important to remark that this study gives perspectives for promising applications and exploitation of the LPG technology for innovative multi-parametric sensing in high radiation environments.

## Methods

### LPG sample fabrication

The sample under analysis was fabricated through a point to point technique, by means of a KrF pulsed excimer laser (LightBench 1000, Optec, Belgium) operating at the wavelength of 248 nm^36^, with pulse width of 5–6 ns. The system includes a motorized rectangular aperture (MRA) mask, characterized by horizontal and vertical dimensions separately selectable in the range 0 ÷ 2500 μm. The spot size of the focused beam on the target is defined by the sizes of the selected MRA and by the demagnification of the focusing objective. During the experiment, the demagnification coefficient was 10:1. A CCD camera provides a real-time monitoring of the laser operation on the sample by a dedicated monitor. At the same time, it provides a real-time control of the sample positioning at the focal distance from the objective which can be adjusted by proper micro-positioning system acting on the vertical position of the focal lens. With regard to the sample positioning, the micromachining system is equipped with an additional X-Y micro-positioning system with resolution of 1 μm and maximum excursion of 10 cm for each axis. It is worth noting that the laser fluence must be properly selected during excimer laser micromachining process. The used system allows controlling the laser fluence on the target by fixing the output laser energy between 0–20 mJ and by acting on an external energy regulator leading to the possibility to finely control the amount of output laser energy reaching the sample^42^. In order to proceed with the grating realization, the fiber was mounted on a customized automatic rotation stage. In particular, the rotation stage consists of two rotating chucks on which the two fiber terminations are fixed allowing a uniform rotation of the fiber itself during the writing procedure. This method guarantees the realization of a uniform refractive core index modulation along the azimuthal fiber axis and, as consequence, the optical spectra are independent on the light polarization. Moreover, both the rotation stage and the laser action are completely controlled and synchronized by a personal computer in order to select the grating pitch (translation stage step and MRA dimension), the grating length (number of irradiated points) and the induced refractive index change (number of laser pulses per point and fluence).

### Irradiation set-up

**Figure 10:**
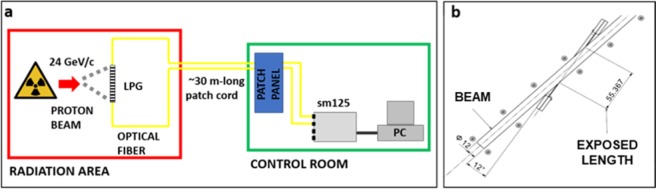
(**a**) Schematic of the experimental set-up used in the IRRAD proton irradiation facility at CERN; **(b)** position of the LPG support with the respect of the proton beam.

A sketch of the experimental set-up in the IRRAD proton facility at CERN is presented in Fig. 10(a). An uncoated LPG sample inscribed in a B-Ge co-doped fiber by means of an excimer laser was installed in a temperature-controlled radiation area, mounted on a designed ad-hoc support to keep the sensor under constant pre-strain conditions for the full duration of the experiment. The holder was positioned at an angle of 12° in respect to the proton beam direction, as shown in Fig. 10(b), thus ensuring to be fully exposed to the beam itself (beam size of ~12 mm FWHM, exposed fiber length of ~55 mm). The interrogation system, represented by a sm125 Micron Optics interrogator featuring a 80 nm wavelength bandwidth in the range [1510–1590] nm, was placed in the control room of the facility, a non-radioactive zone accessible during the irradiation. The sensor under analysis was subjected to a fluence of ~4.4·10^15^ p∙cm^−2^ ± 7%, which corresponds to a proton flux of about 0.8·10^9^ p∙cm^−2^∙s^−1^.
